# Correlation of Prostate-Specific Antigen (PSA), Prostate Volume, and Histology in Ugandan Males

**DOI:** 10.7759/cureus.82937

**Published:** 2025-04-24

**Authors:** Joel Musinzi, Timon M Sseruwagi, Sharon Kalanda, Nicole Lewis, Catherine Lewis

**Affiliations:** 1 General Practice, St. Joseph’s Hospital Kitovu, Masaka, UGA; 2 Orthopaedic Surgery, Kampala International University, Ishaka-Bushenyi, UGA; 3 General Surgery, Kampala International University, Ishaka-Bushenyi, UGA; 4 Radiology, St. Joseph's Hospital Kitovu, Masaka, UGA; 5 Medical Education, Quillen College of Medicine, East Tennessee State University, Johnson City, USA; 6 Surgery, Quillen College of Medicine, East Tennessee State University, Johnson City, USA

**Keywords:** prostate biopsy, prostate cancer, prostate‑specific antigen, prostate volume, uganda

## Abstract

Background

Prostate cancer is the most common malignancy in African men and is increasing in low- and middle-income countries. Prostate-specific antigen (PSA) is a tumor marker for prostate cancer. However, PSA alone does not provide sufficient sensitivity and specificity in assessing prostate malignancy. Previous studies have demonstrated a correlation between age, PSA, and prostate malignancy.

Objective

We sought to determine the correlation between age, PSA, prostate volume (PV), and histology.

Methods

We conducted a retrospective review of male patients at a rural Ugandan hospital who had undergone prostate biopsy. Age, presence of lower urinary tract symptoms (LUTS), PSA, and PV were recorded. A univariate logistic regression model was used to test the probability of PSA, PV, and age to predict benign or malignant prostate histology. A *p-*value of <0.05 was considered statistically significant.

Results

PSA and age were shown to be significant predictors of histology. PSA values >78 ng/mL were also shown to be predictive of malignancy. PV was not a significant predictor of malignant prostate histology.

Conclusions

Our analysis demonstrates that increasing age and elevated PSA levels are significant predictors of prostate malignancy. Larger analyses are needed to determine the correlation between age, PSA, PV, and histology.

## Introduction

Prostate cancer is the most common non-skin cancer diagnosed in men and the second leading cause of cancer mortality following lung cancer [1,2]. Globally, approximately 20% of 100,000 adult men die per year from prostate cancer [3], and African men are disproportionately affected [4]. In Uganda, the incidence of prostate cancer ranges from 35 to 39 per 100,000 [4-6]. The most common predisposing risk factors are age, ethnic background, cardiovascular comorbidities, environmental exposure to chemicals or ionizing radiation, family history, and diet [7-9]. Prostate cancer has been diagnosed in asymptomatic patients or in those with lower urinary tract symptoms (LUTS) such as micturition, difficulty starting urination, increased frequency, and nocturia [4,9]. Most of these symptoms may also be associated with benign prostatic hyperplasia (BPH), which may lead to a late diagnosis of prostate cancer presenting with local invasion and distant metastasis [2,4,9].

Prostate-specific antigen (PSA) is a tumor marker for prostate cancer. Although specific for the prostate, it is not disease-specific as it can be elevated in both benign and malignant processes. PSA can be elevated in BPH, prostatitis, urinary retention, and malignant disease [10-13]. Higher PSA values have also been observed in people of African ancestry [7]. There is no specific PSA value that is associated with carcinoma. Approximately 10% of males have a PSA >10 ng/mL but may or may not have prostate cancer [11]. The inability of PSA values to distinguish between prostate cancer and benign conditions makes it more useful for monitoring prostate cancer treatment rather than as a diagnostic tool [13].

Previous studies have demonstrated a positive correlation between PSA and prostate volume (PV) [12,14]. Increasing age has also been shown to be associated with increased PV and PSA [3,11-13]. A definitive relationship between age, PSA, PV, and tumor histology has not been well established. We sought to investigate the correlation between age, PSA, PV, and tumor histology in Ugandan men who had undergone a prostate biopsy.

## Materials and methods

Institutional Review Board (IRB) approval was granted by East Tennessee State University, Johnson City, TN (protocol 0423.15). A retrospective chart review was performed at a rural hospital in central Uganda from January 2019 to February 2023. All patients who had undergone a prostate needle biopsy were included in the study. Patients who had previously undergone treatment for prostate cancer were excluded. Informed consent was waived in accordance with IRB policies and procedures, and de-identified information was compiled from medical records, imaging, and operative reports.

We collected patient age, PSA, PV, and histological results. All quantitative data were expressed as means. Missing data was not included. A univariate logistic regression model was used to test the ability of PSA, PV, and age to predict benign or malignant histology. Variables with a p-value of <0.05 in the univariate model were considered to be incorporated in a multiple logistic regression model. The reliability of the predictive model was assessed with respect to discrimination and calibration. Predictive model discrimination was analyzed by the area under the receiver operating characteristic curve. The model’s calibration was tested with a Hosmer-Lemeshow goodness-of-fit test. All analyses were performed with Stata/SE, version 16.1 (StataCorp LLC, College Station, TX). A two-sided p-value of <0.05 was considered statistically significant.

## Results

A total of 181 patients underwent a prostate biopsy during the study period. The average age was 67.5 years (range, 45-95 years). The average PSA was 59.41 ng/mL. All patients underwent biopsy due to LUTS. For patients whose PV was recorded, the average was 128.7 cc. There were 165 patients whose histology results were characterized as benign or malignant. Of these, 57.5% were benign (n = 95) and 42.4% were malignant (n = 70; Table 1).

**Table 1:** Descriptive statistics

Variable	Mean
Age (years)	67.5
PSA (ng/mL)	59.41
Prostate volume (cc)	128.7
Histology	Number (%)
Benign	95 (57.5)
Malignant	70 (42.4)

A logistic model with histology as the response and PSA, age, and PV as the predictors was performed. There were 55 observations. Both PSA and age were significant predictors (p = 0.007 and p = 0.0357, respectively). PV was not a significant predictor (p = 0.377).

Since PV did not appear to be a significant predictor of histology, it was removed, and a logistic model was fit with histology as the response and PSA and age as the predictors. PSA was a significant predictor (p = 0.00000915). However, age was not a significant predictor (p = 0.669).

**Figure 1:**
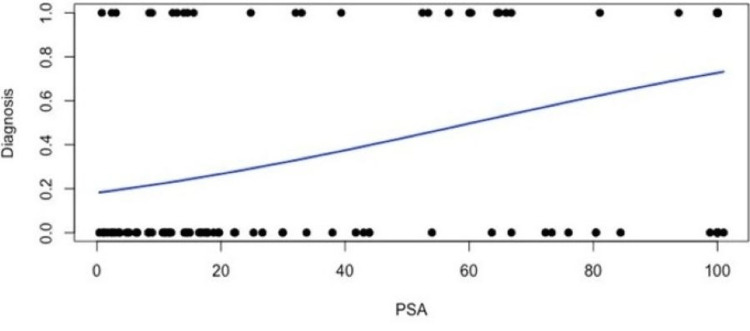
Logistic regression model of prostate-specific antigen (PSA) and histology A plot of the data with the estimated logistic curve superimposed is shown. Since b1 > 0, this indicates the estimated logistic curve is increasing. Here, b1 = 0.024865. Note: e^0.024865^ = 1.025177. We estimated that the odds of a malignant tumor increase by a factor of 1.025177 (by 2.5177%) for each one-point increase in PSA.

Age was then removed from the model, and a logistic model was fit with histology as the response and PSA as the predictor. PSA was a significant predictor of histology (p = 0.00000538). Since b1 > 0, this indicates that the estimated logistic curve was increasing. Here, b1 = 0.024865, where = e^b1^ = e^0.024865^ = 1.025177 (Figure 1).

We estimated that the odds of a malignant tumor increase by a factor of 1.025177 (by 2.5177%) for each one-point increase in PSA. A Hosmer-Lemeshow goodness-of-fit test was performed, and it confirmed the logistic model was a good fit for this data (p = 0.3061). The logistic model is πˆ = eˆ(−1.506564 + 0.024865X)/(1 + eˆ(−1.506564 + 0.024865X)). 

For example, for someone with a PSA level of 74 ng/mL, the estimated probability of a malignant tumor is:

eˆ(−1.506564 + 0.024865 * 74)/(1 + eˆ(−1.506564 + 0.024865 * 74)) = 0.5826.

Therefore, this individual has a 58.26% chance of the tumor being malignant. When assuming the outcomes of malignant and benign are equally likely in the population, one could use the model to predict success (malignant) or failure (benign) for an observation. That is, if the predicted value is 0.5 or greater, you predict success; otherwise, you predict failure. When the PSA level is 74 ng/mL, the predicted value is 0.5826, and the tumor is predicted to be malignant.

## Discussion

Prostate cancer is the most commonly diagnosed cancer in men in South Africa, Nigeria, and Cameroon [15], and African men have been shown to be disproportionately affected as compared to men from other parts of the world [4]. Advanced age, elevated PSA levels, increased PV, and the presence of LUTS have been shown to be associated with an increased risk of developing prostate cancer [3,12]. We sought to further investigate this correlation in a rural community in central Uganda.

The average age of our patients was 67.5 years. This is similar to a meta-analysis in which the majority of African patients with prostate cancer were between 60 and 69 years of age [4]. The incidence of prostate cancer is 60% in men over 65 years of age and has gradually been increasing [8]. Older men have also been shown to have a higher probability of being diagnosed with more aggressive and advanced disease [9]. Our data did not demonstrate that age was a significant predictor of prostate malignancy when only PSA and age were used as predictors of histology.

PSA was initially purified by Wang et al. in 1979 and is produced by the ductal epithelial cells of the prostate gland [11,14,16]. An abnormal digital rectal examination (DRE), the presence of LUTS, and elevated PSA levels frequently lead to prostate biopsies to rule out malignancy [14,17]. In our study, PSA was a significant predictor of malignant prostate histology. In patients with a PSA level of at least 74 ng/mL, there was a 58.26% chance of developing malignancy. Our data is somewhat similar to that of Mainali et al., in which PSA values greater than 20 ng/mL were associated with prostatic carcinoma [13]. PSA has a high positive predictive value for cancer, and men with prostate cancer have been shown to have higher PSA levels compared to those without cancer [11]. However, PSA is also increased in up to 50% of men with BPH [11], making it more useful as a monitor of successful prostate cancer treatment rather than diagnosis [11,13].

Some studies have demonstrated that a smaller PV correlates with a decreased association with prostate cancer. This may be due to lower androgen production, lower intraprostatic growth factor concentrations, or less dihydrotestosterone that leads to a more favorable environment for tumor growth [18]. Al-Khalil et al. also demonstrated an inverse relationship between PV and the aggressiveness of prostate cancer [19]. Larger PVs demonstrated a lower positive biopsy rate for prostate cancer and were associated with a lower Gleason score [19]. PV did not appear to be a significant predictor of histology in our study, likely due to insufficient power to detect a correlation.

PSA does not provide sufficient sensitivity and specificity in assessing prostate disease. PSA values above 4 ng/mL are commonly used to determine the need for prostate biopsy. Values between 4 and 10 ng/mL are considered a gray area in which there is difficulty in making a distinction between BPH and carcinoma [20]. PSA density has been introduced as an additional tool to differentiate BPH from prostate cancer. PSA density is calculated as the total serum PSA divided by the PV [1,20,21]. Previous studies have demonstrated that low PSA density values could eliminate prostate biopsies in men with PSA values in the gray area [20,21]. This signifies the importance of PV as an assessment tool in men with benign and malignant prostate disease. However, our study was not sufficiently powered to determine the correlation between PV, PSA density, and prostate histology.

The impact of prostate cancer in low- and middle-income countries is difficult to describe due to poor cancer registration systems and under-reporting, leading to an unreliable estimation of the global cancer burden [4]. Lack of sufficient data collection and the retrospective nature of data collection are limitations of our study as well. Our study was also not powered enough to determine whether PV plays a role in prostate histology. Due to the small size of our study population, we were also not able to determine Gleason scores for patients diagnosed with prostate cancer and whether elevated PSA values correlated with more aggressive prostate cancer or elevated Gleason scores.

## Conclusions

We present results of a retrospective chart review of all males who underwent prostate biopsy at a rural hospital in Uganda. We found that PSA values and age were significant predictors of prostate histology. Higher PSA values were shown to be predictive of malignancy, while a higher PV was not. Our analysis demonstrates that increasing age and elevated PSA levels are significant predictors of prostate malignancy.
